# In-beam measurement of the hydrogen hyperfine splitting and prospects for antihydrogen spectroscopy

**DOI:** 10.1038/ncomms15749

**Published:** 2017-06-12

**Authors:** M. Diermaier, C. B. Jepsen, B. Kolbinger, C. Malbrunot, O. Massiczek, C. Sauerzopf, M. C. Simon, J. Zmeskal, E. Widmann

**Affiliations:** 1 Stefan-Meyer-Institut für Subatomare Physik, Österreichische Akademie der Wissenschaften, Boltzmanngasse 3, Wien 1090, Austria; 2 Experimental Physics Department, CERN, Genève 23, CH-1211, Switzerland

## Abstract

Antihydrogen, the lightest atom consisting purely of antimatter, is an ideal laboratory to study the CPT symmetry by comparison with hydrogen. With respect to absolute precision, transitions within the ground-state hyperfine structure (GS-HFS) are most appealing by virtue of their small energy separation. ASACUSA proposed employing a beam of cold antihydrogen atoms in a Rabi-type experiment, to determine the GS-HFS in a field-free region. Here we present a measurement of the zero-field hydrogen GS-HFS using the spectroscopy apparatus of ASACUSA's antihydrogen experiment. The measured value of *ν*_HF_=1,420,405,748.4(3.4) (1.6) Hz with a relative precision of 2.7 × 10^−9^ constitutes the most precise determination of this quantity in a beam and verifies the developed spectroscopy methods for the antihydrogen HFS experiment to the p.p.b. level. Together with the recently presented observation of antihydrogen atoms 2.7 m downstream of the production region, the prerequisites for a measurement with antihydrogen are now available within the ASACUSA collaboration.

## Abstract (web summary)

Comparing the ground-state hyperfine structure of antihydrogen to that of hydrogen will provide insights into CPT symmetry in nature. Here the authors report the most precise in-beam measurement of this quantity for hydrogen to demonstrate the viability of ASACUSA's setup to measure it in antihydrogen.

Investigations of the hydrogen atom have been a driving force for the discovery of more profound theories^1^ and contribute to the basis of physics through their prominent influence on the definition of fundamental constants^2^. Most notable from a precision point of view are the recent measurement of the 1S–2S transition via two-photon spectroscopy^3^ and the determination of the hyperfine splitting in hydrogen maser experiments in the early 1970s (refs 4, 5, 6, 7, 8, 9). The achieved absolute (relative) precisions are 10 Hz (4 × 10^−15^) and 2 mHz (1.4 × 10^−12^), respectively. A revival of the interest in hydrogen is founded on prospects of antihydrogen 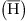 research^10^,^11^. The structure of the simplest anti-atom consisting of a positron bound to an antiproton is predicted to be identical to that of hydrogen, if the combined symmetry of charge conjugation, parity and time reversal (CPT) is conserved. Hence, antihydrogen spectroscopy promises precise tests of the CPT symmetry, which is a cornerstone of the Standard Model of particle physics. A vivid physics programme is currently underway at the Antiproton Decelerator of CERN aiming at spectroscopic^12^,^13^,^14^,^15^,^16^,^17^ and gravity tests^18^,^19^ along with other CPT tests such as the neutrality of antihydrogen^20^,^21^, as well as measurements of the charge-to-mass ratio^22^ and magnetic moment^23^,^24^ of the antiproton.

Among the spectroscopic tests of CPT, the comparison of the ground-state hyperfine structure (GS-HFS) of hydrogen and antihydrogen has the potential to reach the highest sensitivity on an absolute energy scale^25^,^26^,^27^. However, the aforementioned most precise measurement of this quantity for hydrogen was made using a maser^8^. Such a technique is not applicable to anti-matter, which would annihilate with the confining matter enclosure. The measurement proposed by the ASACUSA (Atomic Spectroscopy And Collisions Using Slow Antiprotons) collaboration at the Antiproton Decelerator of CERN therefore makes use of a beam of cold antihydrogen atoms^28^,^29^. In addition to avoiding wall interaction, the actual measurement takes place in a field-free region, ultimately allowing for higher precision compared with the observation of resonant quantum transitions between the hyperfine states in trapped antihydrogen in a high-field environment^30^.

In the present experiment, the Zeeman-shifted hyperfine transitions at various external magnetic field strengths were determined for subsequent extraction of the zero-field value and resulted in


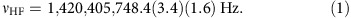


The numbers in brackets are the 1 s.d. (1*σ*) statistical and systematic uncertainties. Added in quadrature the total uncertainty of 3.8 Hz constitutes an improvement by more than an order of magnitude in comparison with the previously achieved best precision by Rabi-type spectroscopy of 50 Hz^31^,^32^. Our result is in agreement within 1 s.d. with the literature value of *ν*_lit_=1,420,405,751.768 (2) Hz, which relies on the more precise hydrogen maser measurements^4^,^5^. In view of the initial goal for antihydrogen GS-HFS of ≲1 p.p.m. relative precision^33^, our hydrogen measurement shows that at this level, systematic uncertainties will be well under control. Our estimate suggests that at least 8,000 antihydrogen atoms of the usually assumed properties will be needed to determine hyperfine transition frequency of antihydrogen 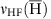 with 1 p.p.m. precision.

## Results

### Spectroscopy principle

**Figure 1:**
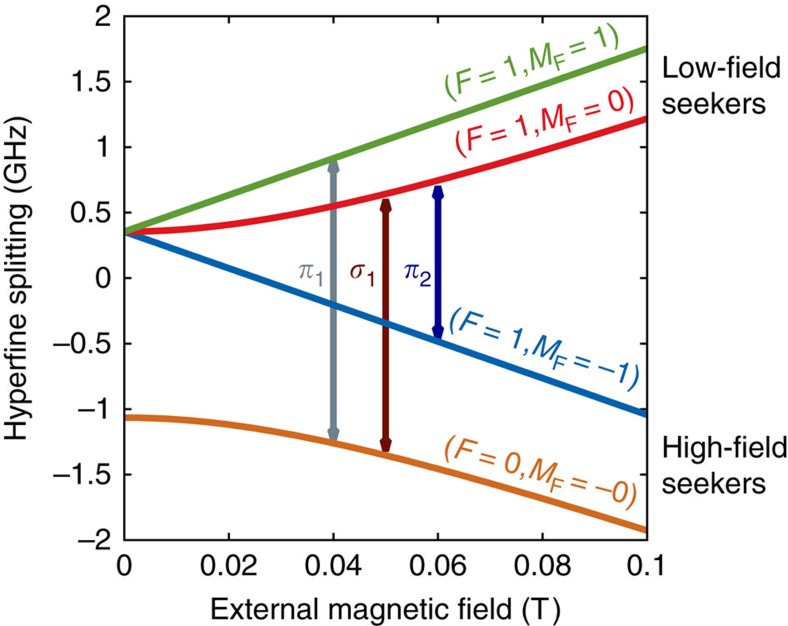
GS-HFS in hydrogen. The Breit–Rabi diagram shows the energy levels in ground-state hydrogen as a function of the strength of an external magnetic field. The four hyperfine states separate into a singlet state and a triplet state, which exhibit different Zeeman shifts. The states with a positive or negative slope are named low- or high-field seekers, respectively. Three possible hyperfine transitions between lfs and hfs are denoted by arrows, the *σ*_1_-transition occurs between the states (*F*=1, *M*_F_=0) and (*F*=0, *M*_F_=0).

Rabi-type magnetic resonance spectroscopy^34^,^35^ applies rotating (or oscillating) magnetic fields to induce quantum transitions and exploits the force of magnetic field gradients on the state-dependent magnetic moment of atoms (or molecules), to spatially separate the atoms in a beam with respect to their quantum states (Stern–Gerlach separation). Typically, magnetic sextupole fields are employed to focus atoms in low-field-seeking states (lfs) and defocus high-field seekers (hfs). In the case of ground-state hydrogen, the hyperfine structure consists of a lower-lying singlet state with total angular momentum quantum number *F*=0 (being proton and electron spin, respectively) and a triplet state *F*=1. As illustrated by the Breit–Rabi diagram in Fig. 1, the triplet state degeneracy is lifted in the presence of a magnetic field. The singlet state and the triplet state with magnetic quantum number *M*_F_=−1 are hfs, whereas the other two states (*F*=1, *M*_F_=0, 1) are lfs. In the present work, the *σ*_1_-transition from (*F*=1, *M*_F_=0) to (*F*=0, *M*_F_=0) has been studied^36^,^37^.

### Experimental setup

**Figure 2:**
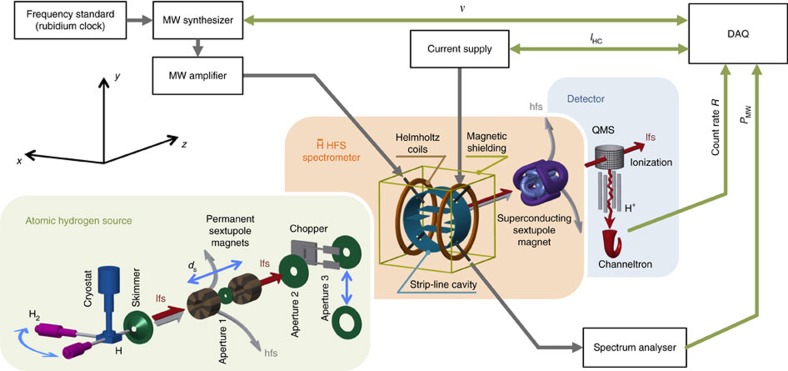
Atomic hydrogen beam setup. Illustration of the three main components of the Rabi-type experimental setup (not to scale). Green panel: the source of cold, polarized and modulated atomic hydrogen. Orange panel: the hyperfine spectrometer of ASACUSA's antihydrogen experiment. Blue panel: the detector. The source consists of a microwave-driven plasma for dissociation of H_2_, a cryostat for cooling the atomic hydrogen beam in a PTFE tubing, two permanent sextupole magnets for polarization and velocity selection, and a tuning fork chopper for beam modulation. The hyperfine spectrometer consists of a state-conversion cavity of strip-line geometry and Helmholtz coils enclosed in a cuboidal Mu-metal shielding followed by a superconducting sextupole magnet for spin-state analysis. The detector employs a QMS for selective mass=1 ion (H^+^) counting after ionization. The count rate is acquired as a function of the driving frequency supplied to the cavity.

The main components of the experiment are a source of cooled and polarized atomic hydrogen, the hyperfine spectrometer of the 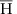 hyperfine splitting (HFS) setup (that is, a microwave cavity and a superconducting sextupole magnet) and a hydrogen detector (*cf*. Fig. 2). The atomic hydrogen source maintains a microwave-driven plasma in a pyrex cylinder to dissociate molecular hydrogen (H_2_→H+H)^38^. Hydrogen atoms are allowed into the first vacuum chamber through a polythetrafluorethylen (PTFE) tubing, which is kept under cryogenic temperatures, to cool the hydrogen atoms and hence reduce their velocity^39^. Two tubing configurations are used in which the plasma-containing pyrex cylinder is either mounted perpendicular to or on axis with the beam. In the first case, a 90° bent tubing assures an efficient and complete interaction of the hydrogen atoms with the cold PTFE surface. In the latter case, a straight tubing keeps the recombinations caused by wall interactions down to a minimum. The cooled atomic hydrogen beam is directed onto a skimmer of 1 mm in diameter and reaches the second, differentially pumped chamber, which houses two permanent sextupole magnets with a pole field of ∼1.3 T at a radius of 5 mm over a mechanical length of 65 mm each^40^. In addition to providing the initial spin-polarization, those sextupole magnets are moveable and feature a midway aperture (aperture 1) to allow for the adjustable selection of a narrow velocity range. As the focusing length depends on the beam velocity, only a certain velocity component is focused onto the aperture and can pass, whereas the off-axis portions of all other components are blocked. The variable distance to the aperture located at half the distance between the sextuple magnets (*d*_s_) therefore selects a velocity component. The resulting velocity distribution is much narrower than a Maxwell–Boltzmann distribution and roughly of Gaussian shape. The spin-polarized and velocity-selected hydrogen beam passes another aperture (aperture 2) and is then modulated by a tuning fork chopper in the next differentially pumped section. The modulation adds time-of-flight measurements to the beam diagnostic tools, as well as suppression of background originating from residual hydrogen via lock-in amplification. Downstream of the chopper, apertures of different diameters (aperture 3) can be installed, to produce different beam sizes at the entrance of the microwave cavity.

The 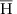 HFS spectrometer has been designed with an open diameter of 100 mm, as a large acceptance is crucial in view of small 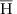 production rates. The amplitude of the oscillating magnetic field *B*_osc_ has to be sufficiently uniform over the large open diameter, to guarantee a trajectory-independent state-conversion probability. This requirement is best met by a cavity of the so-called strip-line geometry^41^,^42^. Two highly transparent meshes confine the microwaves at the entrance and exit of the state-conversion cavity, which are separated by half a wavelength of the hyperfine splitting transition (*L*_cav_∼*λ*_HF_/2∼105.5 mm). A standing wave forms between them and, as a consequence, *B*_osc_ is not constant along the beam propagation direction, causing a double-dip resonance line shape. The origin of this structure is outlined below and explained in detail in the Methods. The cavity length and the beam velocity *V*_H_ define the interaction time of the hydrogen atoms with the microwave field *T*_int_=*L*_cav_/*V*_H_ and restrict the achievable resonance line width to 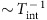. A synthesizer coupled to an external rubidium clock for frequency stabilization produces microwaves, which are fed radially to the cavity via an antenna after amplification. On the opposite side of the cavity, another antenna is used for pick-up and monitoring of the microwave power (*P*_MW_ ∝ *B*_osc_^2^) using a spectrum analyser. Helmholtz coils are mounted onto the cavity to generate a homogeneous external magnetostatic field *B*_stat_, parallel to *B*_osc_, and of several Gauss in magnitude at the interaction region for fine control of the Zeeman splitting. A current source with a relative stability of 20 p.p.m. supplies the Helmholtz coils' current *I*_HC_, which is independently monitored by an amperemeter. *I*_HC_ is directly proportional to *B*_stat_ and turned out to be a better proxy for the magnetic field inside the cavity than a dedicated external magnetic field measurement. The microwave cavity and the Helmholtz coils are surrounded by a two-layer cuboidal Mu-metal shielding to block the Earth's magnetic field, as well as the fringe field of the closely succeeding superconducting sextupole magnet. Owing to the pole strength of up to 3.5 T, this magnet generates sizeable magnetic field gradients despite the large open diameter of 100 mm. The integrated gradient amounts to 150 Tm^−1^ and ensures refocusing of 50 K lfs atoms within a distance of ∼1 m.

The detection of hydrogen suffers from a large background rate and small efficiencies. A crossed-beam quadrupole mass spectrometer (QMS) with a 3 mm opening ionizes beam atoms and residual gas by electron impact and selectively guides protons to a channeltron for efficient single mass=1 ion counting. The small sensitive area and hydrogen ionization efficiency result in detected beam rates of only a few kHz in spite of typical H_2_ flowrates of 1.8 × 10^17^ s^−1^. The background rate is kept at a level of only few tens of kHz by combining two-stage turbo-molecular pumping and non-evaporable-getter pumps, thereby maintaining ultrahigh vacuum conditions (≲5 × 10^−10^ mbar) in the detection chamber. Furthermore, the QMS can be moved two-dimensionally in the plane perpendicular to the beam for optimizing count rates and investigating beam profiles.

### Measurement procedure

The dissociation plasma was operated under stable standard conditions. Before starting frequency scans, the microwave power *P*_MW_ supplied to the cavity was adjusted to yield the largest state-conversion probability by observing a Rabi oscillation. A single measurement cycle was obtained by scanning the frequency once in a random sequence across the desired range. Typically, this included *N*∼39 frequency points distributed over ∼40 kHz. At each frequency point, the channeltron events of the QMS were summed several times for typical intervals of 5–60 s from which an average count rate was retrieved. Such cycles over the frequency range were repeated on average five times with changing random sequences to result in a complete scan at a given *I*_HC_. This was repeated at different values and polarity of *I*_HC_ to yield a set of scans suitable for determination of the field-free hyperfine splitting. The number of *I*_HC_ values per set ranged from 6 to 16. In total, ten such sets have been recorded, which differ in various of the experimental settings and arrangements (*cf*. Table 1).

### Raw data corrections

Initially, a fit as described below was applied to the detected count rates. Two systematic effects were identified in the residuals and corrected for. The first correction compensates slow time drifts. The second correction concerns a type of memory effect, which became evident in an increased likelihood of observing positive or negative residuals if the previous data point was taken at higher or lower count rate, respectively. This indicated that the settling of the hydrogen rate in the detection chamber following a change of the excitation frequency had a non-negligible time constant when compared with the measurement time at each frequency step. These two effects were corrected for at the raw data level and led to an improvement of the fit quality without affecting the extracted *ν*_HF_ values. The application of a random sequence of frequencies in the cycles seemed to suppress systematic impacts of the drift and the memory effect below the statistical sensitivity.

### Analysis

**Figure 3:**
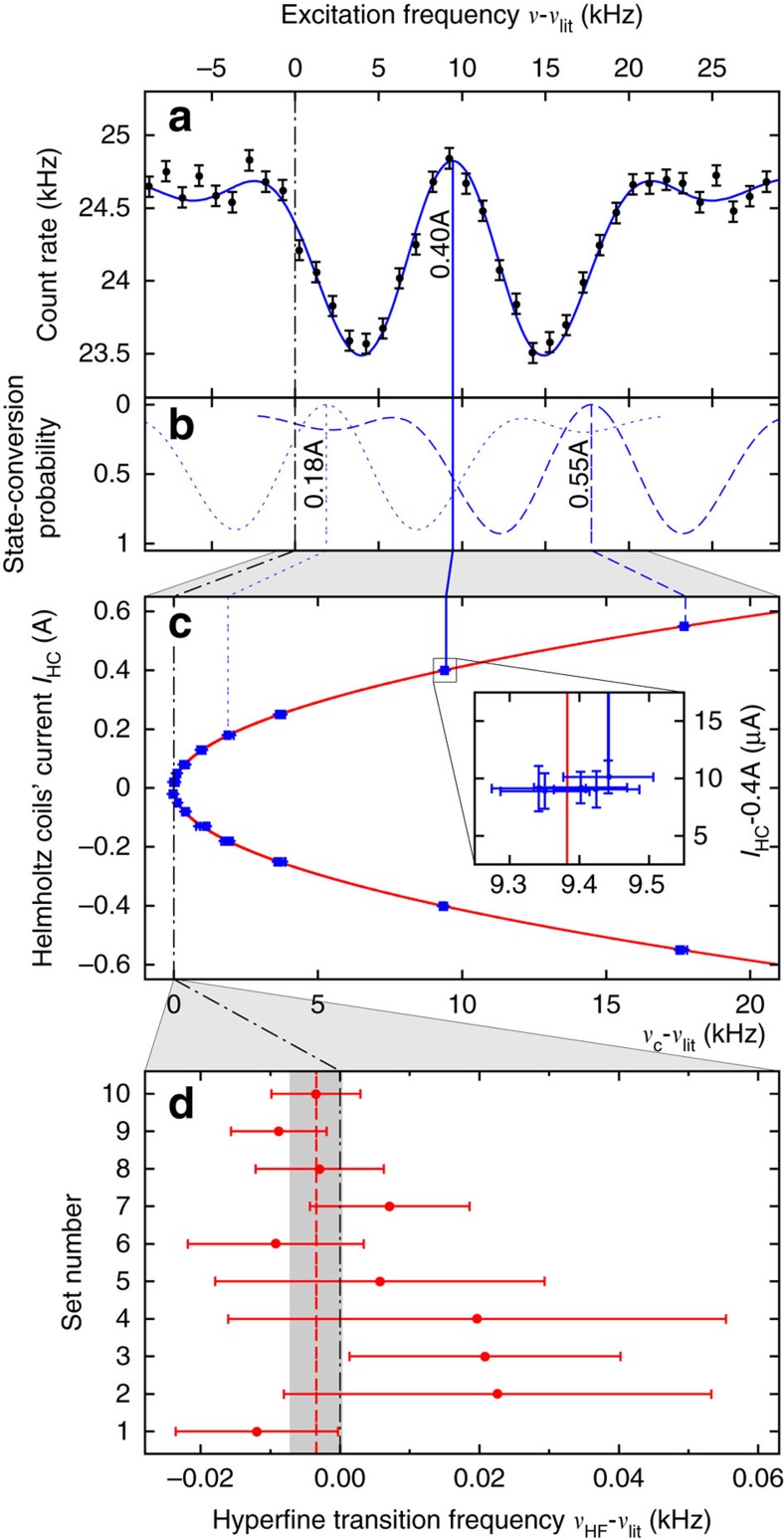
Resonance spectrum and zero-field value extraction. (**a**) Data with Poisson errors of one cycle of set 8 at a Helmholtz coils' current *I*_HC_=400 mA fitted with the resonance curve (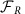, full blue line, see equation (13) in Methods) to extract the central frequency *ν*_c_, which is Zeeman-shifted to values >*ν*_HF_. A dashed-dotted black line at 0 is drawn through all the plots to represent *ν*_lit_. (**b**) State-conversion probabilities 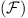 as obtained from the fit 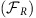 of two other cycles of the same set, but at different settings of *I*_HC_ (dotted blue line 180 mA, dashed blue line 550 mA, data omitted for clarity). (**c**) *ν*_c_ of all 80 cycles of set 8 (16 different values of *I*_HC_, 5 cycles each) plotted against *I*_HC_ for extraction of the zero-field hyperfine splitting *ν*_HF_ using the Breit–Rabi fit function 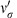 (red line) of equation (4). The inset is a zoom into the group of five cycles at *I*_HC_=400 mA, illustrating the typical size of the frequency and current s.d. of each data point. (**d**) The resulting *ν*_HF_ as deviation from *ν*_lit_ for the 10 sets (red s.d. error bars) and their weighted mean value (dashed red line) with the 1 s.d. total uncertainty as grey-shaded area.

The central frequency *ν*_c_ was extracted from every cycle by a fit to the spectrum as illustrated in Fig. 3a, where the excitation frequency *ν* is given as the difference to *ν*_lit_. The double-dip line shape originates from the sinusoidal dependence of *B*_osc_ along the beam axis, which follows half a cosine period. At the actual transition frequency, the highest count rate between the two dips is observed. The theoretical line shape for a monoenergetic beam is well understood and accurately described within the framework of the two-level system with the interaction Hamiltonian


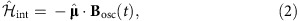


where 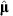 is the magnetic moment operator as defined in equation (9). The time dependence of the magnetic field includes a cos(*πt*/*T*_int_)-term in addition to the microwave oscillations. The resulting equations were solved numerically to obtain the state-conversion probability as a function of the frequency *ν* and amplitude *B*_osc_ of the driving field for a monoenergetic beam. A realistic fit function 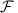 for the measured state-conversion probabilities was obtained by convolution of the shape for monoenergetic beams with a velocity distribution as described in the Methods. Consequently, the fit function could extract the physical parameters *B*_osc_, the mean velocity of the polarized atomic hydrogen beam 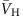 and the width of the velocity distribution *σ*_*V*_ in addition to *ν*_c_ of the transition. Two further fit parameters of less relevant physical content scaled the state-conversion probability to the count rate and correspond to the count rate baseline *R*_0_ and the count rate drop Δ*R* for complete state conversion. In the final analysis, only *ν*_c_ was extracted from every cycle individually. For *B*_osc_, a relation to the monitored microwave power was established based on the complete available data. This enabled individual fixation of this parameter for every set and avoided non-converging fits due to a strong correlation of *B*_osc_ with Δ*R*. For 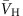 and *σ*_*V*_, a common fit value for a complete set was used, as all settings of direct impact on the beam velocity remained unchanged during data collection of a set.

As illustrated in Fig. 3a, the line shape thus obtained resulted in good fits to the observed count rates at all *I*_HC_ settings with reduced *χ*^2^-values close to unity as summarized in Table 1. The reliability of the fit function was important, as *ν*_c_ could be extracted with typical statistical uncertainties on the order of tens of Hz, whereas the width of the double-dip structure is on the order of tens of kHz. In Fig. 3c, the extracted *ν*_c_ value of each cycle of set 8 are plotted against the Helmholtz coils' current *I*_HC_ at which it was recorded. The Zeeman-shifted frequency of the *σ*_1_-transition *ν*_*σ*_(*B*_stat_) has only a second-order dependence on the static external magnetic field *B*_stat_ as apparent from the Breit–Rabi diagram (Fig. 1) and described by the Breit–Rabi formula^43^


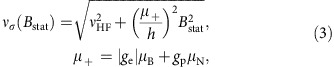


with *μ*_B_=5.7883818012 × 10^−5^ eVT^−1^ and *μ*_N_=3.1524512550 × 10^−8^ eVT^−1^ being the Bohr and nuclear magneton, respectively, *g*_e_=−2.00231930436182 and *g*_p_=5.585694702 (ref. 2) being the *g*-factors of the electron and proton, respectively, and *h*=2*πħ* the Planck constant. To extract the zero-field hyperfine transition frequency *ν*_HF_, a fit function 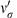 was required, which used *I*_HC_ as a variable. A factor *k* converting *I*_HC_ to a magnetic field and a residual field *B*_res_ at *I*_HC_=0 added two further fit parameters and established a linear relation to *B*_stat_, which enters the Breit–Rabi formula


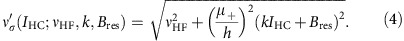


The notation for the fit function separates the variable from the parameters by a semicolon. The zero-field values *ν*_HF_ as obtained via this Breit–Rabi fit are plotted in Fig. 3d as the deviation from *ν*_lit_.

### Systematic tests

The following experimental arrangements and conditions have undergone changes for the ten sets (summarized in the top part of Table 1). The beam velocity varied due to different settings of *d*_s_ and the temperature of the PTFE tubing. The first three sets operated with the straight PTFE tubing, then the bent tubing was used. The need for an improved monitoring of *I*_HC_ and the advantage of a faster data acquisition scheme based on the total count rate instead of the lock-in amplifier signal became evident in a preliminary evaluation of the first three sets and motivated the additional changes at that stage. Two opening diameters for aperture 3, resulting in different beam sizes at the entrance of the cavity, were also investigated. This is of special interest as an even larger beam diameter is expected for the 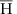 HFS spectroscopy. In addition, the last four sets were performed with a second cavity of the same but slightly upgraded design. Three aspects were only changed for individual sets. For set 7 only one instead of two layers of magnetic shielding were used, for set 3 the superconducting sextupole was operated with a larger magnetic field strength leading to a shorter focal length and for set 4 the direction of the static magnetic field (Helmholtz coils) was not reversed.

The obtained results for *ν*_HF_ of the ten sets by first fitting all cycles in a set using the fit function (12) and second the Breit–Rabi fit (4) are presented in Fig. 3d. In addition, the average reduced *χ*^2^ of all fits to cycles within a set and the reduced *χ*^2^ of the Breit–Rabi fit are given in Table 1. On the level of the achieved statistical precision, no significant dependence of the ten results on any of the changed experimental conditions could be found. This justified to combine the ten individual results into one weighted mean value. Our final result deviates from the literature value by *ν*_HF_−*ν*_lit_=−3.4 Hz with a total uncertainty of *σ*_tot_=3.8 Hz, which corresponds to a relative precision of 2.7 p.p.b. The mean value is shown in Fig. 3d as the dashed red line and the total 1*σ* uncertainties as the grey-shaded area.

The fit parameters *B*_osc_, 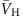 and *σ*_*V*_, which were fixed to a common average value for each set, were varied to assess the potential systematic uncertainties originating from the fit procedure. The complete analysis was repeated six times with setting each of the three parameters individually to its lower and upper 1*σ* boundary. The observed shifts of *ν*_HF_ for each parameter are listed in Table 2. However, those three values added in quadrature yielded 0.06 Hz and present a negligible systematic uncertainty. The rubidium clock, which served as frequency standard, supplied a 10 MHz reference signal to the microwave synthesizer. A calibration was performed several months after the measurement campaign and revealed a shift of 11.4 mHz or equivalently 1.14 p.p.b. This corresponds to 1.6 Hz for *ν*_HF_, which is less than half the total statistical error. Given the timespan between the measurement and the calibration and the unknown evolution in time of the shift, it was not corrected for but instead conservatively added as a 1*σ* systematic uncertainty. A correction would have brought the central value closer to the literature value by roughly half a s.d. Table 2 summarizes the error budget.

## Discussion

In the antihydrogen Rabi spectroscopy proposal^33^, the first-stage precision goal is ≲1 p.p.m. According to the Standard Model Extension framework^25^, the absolute precision is more decisive, to quantify and compare the sensitvity level of CPT tests. A p.p.m. measurement of the GS-HFS of antihydrogen corresponds to kHz frequencies or peV energies. This would already be several orders of magnitude better than the ∼2 neV precision of the kaon–antikaon comparison^44^ and competitive with the best achievable test using the 1S-2S transition^17^. In addition, at the level of ∼40 p.p.m. the antiproton structure becomes relevant in the calculation of the hyperfine structure of antihydrogen through the Zemach and nuclear polarizability corrections^45^. Therefore, together with independent measurements of the antiproton magnetic moment^23^,^24^, a p.p.m. measurement of the GS-HFS of antihydrogen would give access to the electric and magnetic form factors of the antiproton. The result presented here on hydrogen shows that systematic uncertainties can be controlled much beyond the p.p.m. level. The data can also be used to assess the prospects for antihydrogen hyperfine spectroscopy. As first documented in ref. 46, three main terms quantitatively describe the precision with which a fit parameter of a resonance spectrum line shape can be determined. They relate to the signal-to-noise ratio, the number of data samples per line width and a line-shape-dependent factor. If the fit parameter of interest is the central frequency, the last term primarily expresses the resolution of the method. We adapt this formula to our case:


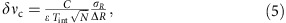


where the signal is identified with the count rate drop for complete state conversion Δ*R* and the noise with the average error bar *σ*_*R*_ of a data point. Instead of the data sample density, the dimensionless number of frequency data points *N* enters as the inverse square root. The interaction time *T*_int_ appears explicitly, as it is inversely proportional to the line width, which is a measure for the resolution. The dimensionless constant *C* should be unity and is inserted to test the relation. All remaining quantities are collected in a dimensionless line-shape-dependent factor *ɛ*, which for instance accounts for the effects of different velocity distributions. Formula (5) is verified using the present hydrogen data. Apart from a consistent underestimation by a factor of *C*∼1.07, the precision is predicted reliably. More details can be found in the Methods.

The prospects of an antihydrogen GS-HFS measurement using the *σ*_1_-transition can now be assessed by formula (5) making assumptions for yet unknown beam properties. Under Poisson statistics, *σ*_R_ will be proportional to the square root of the total number of 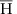 events registered at the annihilation detector 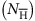, which includes lfs of ground-state antihydrogen, as well as excited antihydrogen and false identifications from cosmic radiation or upstream annihilations. The number of excited states will be limited in the antihydrogen experiment by field ionization of Rydberg atoms down to the main quantum numbers *n*=12 (ref. 47). Given the typical decay times of states with *n*<12, most 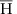 atoms should reach the cavity in the ground state. Conservatively, we assume that half of the atoms remain in excited or meta-stable states. According to statistical weights, another half of the lfs will be in the state (*F*=1, *M*_F_=1). For a completely polarized beam, this remaining quarter of the total detected events would correspond to Δ*R*. We assume a polarization effect by the cusp magnetic field gradients of *p*=1/3 (refs 48, 49), which leads to a further reduction of Δ*R* proportional to 2*p*/(*p*+1). The annihilation detector, which consists of a central calorimeter^50^ and a double layer hodoscope for pion tracking^51^, suppresses background from false identifications to negligible levels. For the last term of formula (5), we find


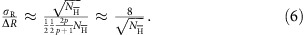


A signal-to-noise ratio of 2 or 3 will require 250–600 events for each data point. The ASACUSA collaboration aims at reaching an antihydrogen temperature below 50 K. We assume a Maxwell–Boltzmann velocity distribution of the emerging beam as the worst case scenario, as other selection mechanisms are expected to lead to a smaller velocity spread. The most probable velocity of antihydrogen with this temperature is 909 ms^−1^; hence, the estimated interaction time is 116 μs. The correction coefficient *ɛ* can reach values of ∼1.2 by concentrating on the central peak of the resonance instead of resolving the full double-dip structure as discussed in the Methods. In addition, the central peak can be encompassed with a low number of frequency points of *N*∼8. Inserting all numbers into formula (5) result in a statistical precision of *δν*_c_=1.38 kHz or ≲1 p.p.m. with 2,000 detected 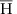 events (and *δν*_c_=0.89 kHz with 4,800 events). For a zero-field determination as demonstrated in the present measurement on hydrogen, a minimum of four resonances will be required as the Breit–Rabi fit (4) has three parameters. Consequently, an estimated minimum of 8,000 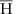 events will be required to determine 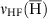 with a precision of 1 p.p.m.

## Methods

### Resonance line shape

The *σ*_1_-transition in ground-state hydrogen is driven by an external microwave field, which is generated in a strip-line cavity and takes the form


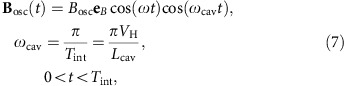


where **e**_*B*_ is the unit vector pointing in the direction of the magnetic field (*z* axis in the frame of the atoms, *x* axis in the coordinate system of the experiment) and *ν*=*ω*/2*π* is the applied microwave frequency. The term cos(*ω*_cav_*t*) describes the changing amplitude of the magnetic field in the cavity along the beam propagation direction. *T*_int_ is the interaction time, which in turn follows from the hydrogen beam velocity *V*_H_ and the length of the cavity *L*_cav_.

The small external magnetic field is aligned parallel to the oscillating magnetic field, which only for the *σ*_1_-transition leads to non-vanishing matrix elements. In addition, the Zeeman shift separates the ground-state hydrogen sub-levels by more than the observed resonance width. Therefore, the transition dynamics is well described within the framework of the two-level system


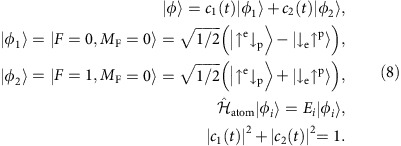


To obtain the time evolution of this system under the influence of the oscillating magnetic field, the Hamiltonian needs to be extended by the interaction 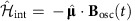 with


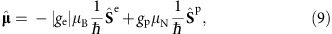


for hydrogen. Here, 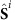 are the spin operators acting on the electron or proton spinor as indicated by the superscript. An analytical solution can be found for conventional Rabi experiments, where the oscillating (or rotating) magnetic field has a constant amplitude *B*_osc_ and does not include the term cos(*ω*_cav_*t*). If the system is initially prepared purely in state 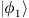, then the conversion probability 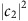 of finding it after a given interaction time *T*_int_ in the second state 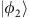 depends on the strength of *B*_osc_ and the detuning Ω_D_=*ω*−*ω*_12_ with *ħω*_12_=*E*_2_−*E*_1_:


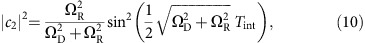


where Ω_R_ is the Rabi frequency, which is proportional to the amplitude of the oscillating magnetic field. The relation for the *σ*_1_-transition is


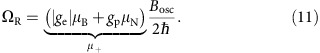


**Figure 4:**
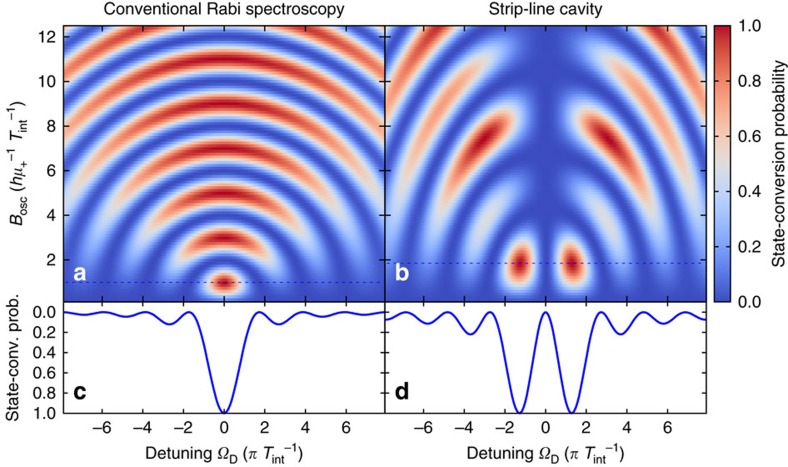
State-conversion maps for driving strength and detuning. Comparison of the state-conversion probabilities as a function of the detuning *Ω*_D_ (in units of 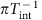) and the amplitude of the oscillating magnetic field *B*_osc_ (in units of 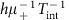) for the case of conventional Rabi spectroscopy (**a**) and when using a strip-line cavity (**b**) to drive the transition. Both cases refer to a monoenergetic beam, which translates to a fixed interaction time *T*_int_. The dashed horizontal line indicates the required driving strength to reach the first complete state conversion. The plots (**c**,**d**) are projections of the state-conversion probabilities at the dashed lines and show the ideal (that is, monoenergetic) line shapes observed as count rate drops in the Rabi experiments.

Including the term cos(*ω*_cav_*t*) requires numerical methods to determine the state-conversion probability. Figure 4 shows a comparison of 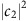 as a function of the detuning Ω_D_ and the driving strength *B*_osc_ of conventional Rabi spectroscopy and the strip-line cavity designed for the antihydrogen experiment. The latter case features the distinct double-dip structure with vanishing effects at the actual transition frequency. For a given interaction time *T*_int_, the best precision is achieved with the first full-state conversion in both situations. For the conventional case, this corresponds to a so-called *π*-pulse, indicating that the condition Ω_R_·*T*_int_=*π* is satisfied or alternatively 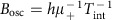. The double-dip resonance reaches the first full-state conversion when applying a somewhat stronger oscillating magnetic field 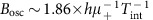.

From a two-dimensional (2D) map as shown in Fig. 4, fit functions of the state-conversion probabilities for a monoenergetic beam can be derived with *ν*_c_, the strength of *B*_osc_ and the hydrogen beam velocity *V*_H_ as fit parameters. This was realized by constructing a 2D spline interpolation 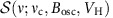 to the numerically generated state-conversion probabilities at discrete points. A more realistic resonance line shape is then obtained by including the effect of the velocity distribution of the hydrogen beam, which translates to a not sharply defined interaction time *T*_int_. It is noteworthy that both axis of the 2D maps are normalized to 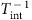. Therefore, on an absolute scale for Ω_D_ and *B*_osc_, a change of *T*_int_ is equivalent to a 2D zooming of the state-conversion probability map. The roughly Gaussian velocity distribution of the hydrogen beam after passage of the polarizing and velocity-selecting permanent sextupole magnets is approximated by binomial coefficients for a discrete numerical realization of the convolution


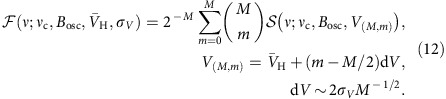


**Figure 5:**
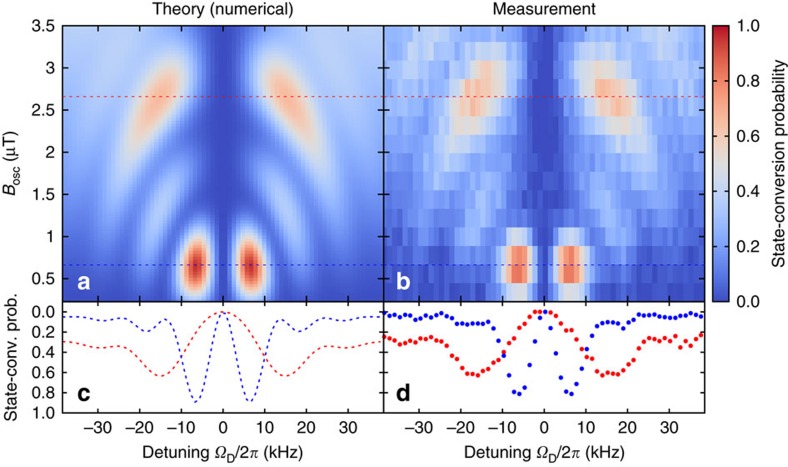
State-conversion maps with a velocity spread. Comparison of theoretical and measured state-conversion probabilities as a function of the detuning *Ω*_D_/2*π* in units of kHz and the amplitude of the oscillating magnetic field *B*_osc_ in units of μT. The theoretical map (**a**) includes the effect of a Gaussian-like velocity distribution with 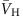
=1,060 m s^−1^ and *σ*_*V*_=95 m s^−1^. The measurement (**b**) was taken setting a large distance between the permanent sextupole magnets of *d*_s_=115 mm, as it has been used for the sets 1 and 10. The blue and red dashed horizontal lines indicate the driving strengths, where the first and second full-state conversion would be reached in the case of a monoenergetic beam. The plots (**c**,**d**) are projections of the state-conversion probabilities at the dashed lines showing good agreement between theory and measurement. Frequency spectra across the narrow double-dips at the first state conversion yield the highest precision.

The result of a convolution with such a velocity distribution is illustrated in Fig. 5 and compared with a measured map. The present analysis used *M*=6, because choices of *M*>6 did not change nor improve the fit results. For completeness, two more fit parameters were needed. To scale the state-conversion probability, which is a number between 0 and 1, to the observed count rates, a count rate baseline *R*_0_ and a count rate drop for complete state conversion Δ*R* were introduced


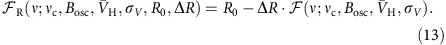


### Investigations on the precision

The precision with which a parameter *x*_*i*_ of a line shape 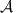 can be determined by minimum least square fitting is related to the noise *σ* of the resonance spectrum by


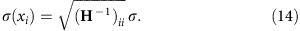


The elements of the matrix **H** are given by


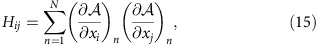


where the summation is taken over the *N* data points^46^. Equation (5) can be derived from the relations above and yields the following expression for the line-shape-dependent correction factor


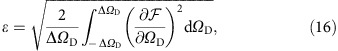


where 2·Δ*Ω*_D_ gives the covered scan range in units of 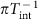 and the sum has been simplified to a symmetric integral.

**Figure 6:**
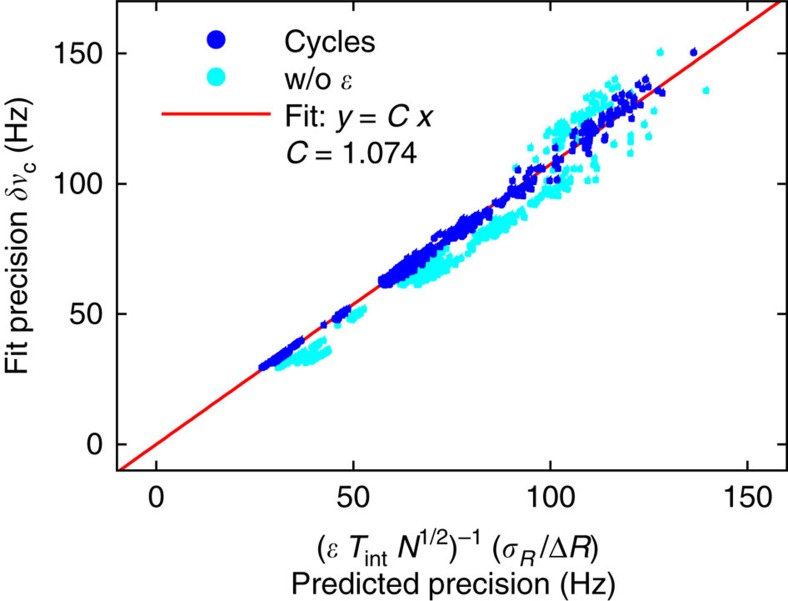
Predictability of the precision. The precision of the central frequency *ν*_c_ as obtained from a line shape fit to a cycle is plotted against the quantities, which enter the proportionality formula (5). A line fit (red line) through the origin extracts the proportionality constant *C*, which should be equal to 1. All 545 cycles recorded within the ten sets are included in this plot. The dark and bright blue dots depict the situation when the correction factor *ɛ* is included or omitted, respectively.

Figure 6 visualizes the test of equation (5) using the present data by plotting the precision extracted by the fit against the predicted precision with and without inclusion of the correction factor *ɛ*. The data set covers all 545 cycles of the 10 recorded sets. The correction for line-shape-dependent effects by *ɛ* improves the reliability of the predicted precision and plays a more important role when making projections for antihydrogen spectroscopy, where different beam properties have to be expected. The slope of a line fit through the origin yields the dimensionless constant *C*.

**Figure 7:**
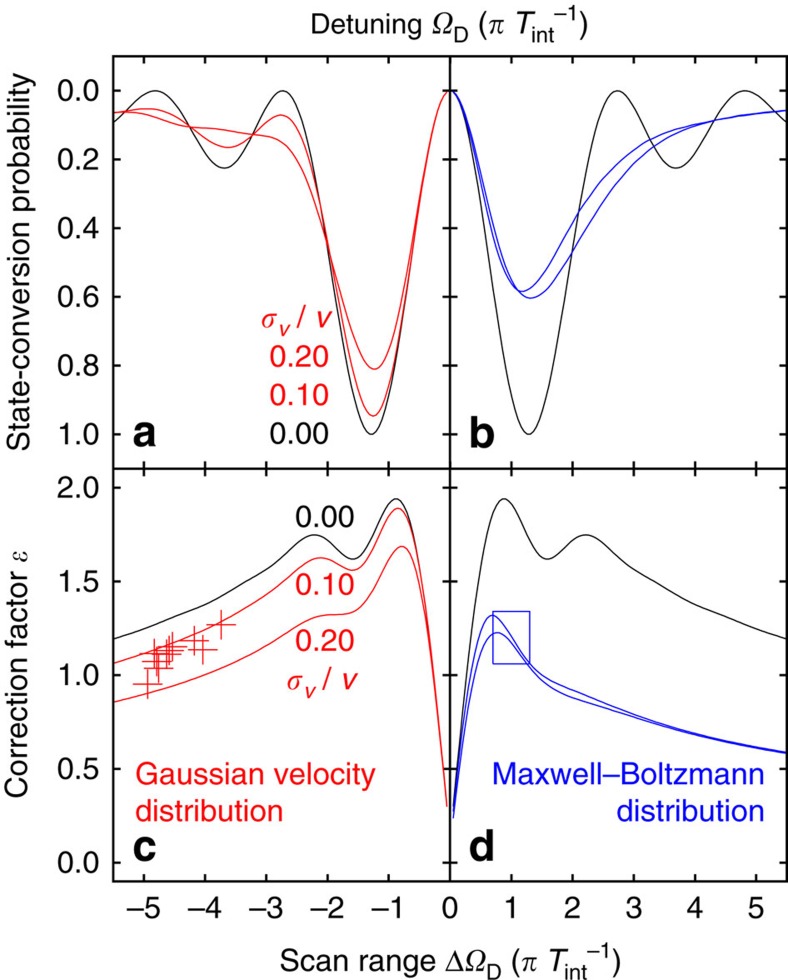
Lineshape-dependent correction factors. The theoretical conversion probabilities 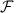 for a monoenergetic beam (black) is compared in (**a**) with the case of Gaussian distributed beam velocities of various widths (red) and in (**b**) with the Maxwell–Boltzmann distributed beam velocities (blue). The corresponding correction factors *ɛ* as defined in equation (16) are shown in (**c**,**d**) for the compared velocity spreads, respectively. For (**a**,**b**) the *x* axis is the detuning *Ω*_D_, whereas for (**c**,**d**) it is the scan range Δ*Ω*_D_, both in units of 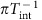. The crosses in (**c**) represent the correction factors for the ten sets. The correction factor for the estimate of required antihydrogen events is marked by a rectangle in (**d**).

Figure 7 compares the line shapes 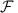 and correction factors *ɛ* of a monoenergetic beam to Gaussian and Maxwell–Boltzmann distributed beams. The crosses mark the correction factors determined for the ten sets, which depend on the chosen scan range, on the amplitude of the oscillating magnetic field *B*_osc_ and on the relative width of a Gaussian velocity distribution 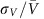, as determined by the fit. For the resonance line shape of a Maxwell–Boltzmann distributed beam, the correction factors are generally smaller, which indicates reduced precision. The curves for the Maxwell–Boltzmann distributed beam show the cases, when *B*_osc_ is optimized for either the most probable or root-mean-square velocity. For the antihydrogen measurement with limited count rates, the scan range dependence of the correction factor reveals that it will be beneficial to restrict the resonance scan to the central peak.

### Data availability

The data sets generated and analysed during the current study are available from the corresponding author on reasonable request.

## Additional information

**How to cite this article:** Diermaier, M. *et al*. In-beam measurement of the hydrogen hyperfine splitting and prospects for antihydrogen spectroscopy. *Nat. Commun.* **8,** 15749 doi: 10.1038/ncomms15749 (2017).

**Publisher's note**: Springer Nature remains neutral with regard to jurisdictional claims in published maps and institutional affiliations.

## Supplementary Material

Peer Review File

## Figures and Tables

**Table 1:** Parameters of the data sets.

**set**	**1**	**2**	**3**	**4**	**5**	**6**	**7**	**8**	**9**	**10**
PTFE tubing	Straight	Straight	Straight	90 deg.	90 deg.	90 deg.	90 deg.	90 deg.	90 deg.	90 deg.
Cryostat temperature (K)	23	16	100	50	50	50	50	50	50	50
*d*_s_ (mm)	115	35	91	21	21	16	16	16	16	115
Cavity	# 1	# 1	# 1	# 1	# 1	# 1	# 2	# 2	# 2	# 2
Precise monitoring of *I*_HC_	No	No	No	Yes	Yes	Yes	Yes	Yes	Yes	Yes
Supercond. sextupole (A)	350	350	400	350	350	350	350	350	350	350
Beam diameter (mm)	8	8	8	8	8	8	8	8	22	22
Shielding layers	2	2	2	2	2	2	1	2	2	2
*I*_HC_ Polarity	±	±	±	+	±	±	±	±	±	±
Number of scans	8	6	6	10	12	12	16	16	12	12
Number of cycles	23	46	26	50	60	60	80	80	60	60
Frequency data points *N*	41	21	26	39	39	39	39	39	39	39
Acqu. time/data point (s)	60	40	40	5	5	5	5	5	5	5
*V* (m s^−1^)	1,066 (1)	962 (2)	1,152 (2)	888 (2)	857 (3)	883 (2)	933 (2)	922 (1)	1,049 (1)	1,131 (1)
*σ*_*V*_ (m s^−1^)	152 (2)	145 (3)	156 (2)	160 (2)	184 (2)	139 (2)	124 (2)	129 (2)	183 (1)	149 (1)
*B*_osc_ (10^−7^ T)	6.86 (0.01)	6.49 (0.01)	8.14 (0.01)	5.73 (0.01)	5.81 (0.01)	5.78 (0.01)	6.70 (0.03)	6.28 (0.05)	6.54 (0.03)	6.93 (0.03)
*R*_0_ (Hz)	27,088 (232)	24,420 (576)	26,517 (234)	26,998 (458)	20,889 (237)	26,118 (84)	23,100 (194)	24,825 (225)	56,390 (2,806)	31,584 (1,724)
Δ*R* (Hz)	891 (30)	476 (43)	1,112 (29)	1,471 (71)	795 (47)	1,484 (50)	1,126 (42)	1,401 (51)	4,499 (907)	3,284 (154)
Av. *χ*^2^/n.d.f. of res. curves	2.6 (0.7)	1.6 (0.5)	1.9 (0.5)	1.2 (0.3)	1.0 (0.3)	1.2 (0.2)	1.1 (0.3)	1.1 (0.2)	1.9 (0.4)	1.9 (0.4)
*B*_res_ (10^−7^ T)	4.0 (1.0)	3.9 (2.6)	2.3 (2.0)	11.9 (19.1)	5.4 (1.9)	5.7 (1.0)	3.1 (1.3)	3.5 (1.1)	2.5 (0.5)	2.7 (0.5)
*k* (10^−5^ T/A)	45.83 (0.02)	45.47 (0.16)	45.70 (0.14)	45.87 (0.37)	45.76 (0.06)	45.80 (0.03)	45.85 (0.04)	45.90 (0.03)	45.90 (0.02)	45.89 (0.02)
*χ*^2^/n.d.f. of Breit–Rabi fit	17.5/21	38.6/44	41.3/24	70.0/47	65.3/57	48.5/57	101.0/77	83.2/77	75.0/57	55.6/57
*ν*_HF_−*ν*_lit_ (Hz)	−12.0 (10.6)	22.6 (22.2)	20.8 (19.4)	19.7 (46.7)	5.7 (23.9)	−9.2 (12.8)	7.1 (11.4)	−2.9 (9.2)	−8.8 (6.9)	−3.5 (6.4)

Comparison of the ten sets. The four blocks of rows summarize experimental conditions, statistics of the data acquisition, average fit parameter of cycles together with the average reduced *χ*^2^ from applying fit-formula (13), and finally the fit parameters and the corresponding reduced *χ*^2^ from applying the Breit–Rabi fit (4).

**Table 2:** Error budget.

**Contribution**	**1*****σ*** **s.d. (Hz)**
Systematic error	
Frequency standard	1.62
Common fit parameters	
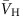	0.05
*σ*_*V*_	0.03
*B*_osc_	0.02
Systematic error total (*σ*_sys_)	1.62
Statistical error (*σ*_stat_)	3.43
Total error (*σ*_tot_)	3.79
