# Supplementary material for: In-beam measurement of the hydrogen hyperfine splitting and prospects for antihydrogen spectroscopy
Source: Nat Commun. 2017 Jun 12;8:15749. doi: 10.1038/ncomms15749 (PMC5472788; doi:10.1038/ncomms15749)
Supplement: Peer Review File [file ncomms15749-s1.pdf]

Reviewer #1 (Remarks to the Author):

I have been asked to review the article “In-beam measurements of the hydrogen hyperfine splitting – towards antihydrogen spectroscopy” for Nature Communications. Please find it here.

The paper makes a number of well supported major claims about the research. A great deal of progress has been made to prepare for antihydrogen spectroscopy by the ASACUSA collaboration. As all experiments with antimatter atoms, it will be extremely challenging to achieve sufficiently high signal to noise ratios for precise spectroscopy due to the small number of atoms that can be produced, limited by antiproton availability. This warrants the preparation and testing of such apparatus with matter atoms wherever possible as described here. The apparatus has been developed over a long period and with a high level of care and planning to achieve its present state. The authors are applauded for this effort and congratulated on their progress in developing this novel apparatus for antihydrogen measurements.

The work results in a comparison of spectroscopic measurements in hydrogen intervals at the ppb level with the much high precision (ppt and ppq level) achieved in apparatus not suited for antihydrogen spectroscopy and referenced within. The present results are in reasonable agreement and hence conclude that there are no systematic shifts evident at the ppb level. Although antihydrogen production will be with a very different technique and will result and many orders of magnitude reduced signal counts, these results will not be systematically shifted and hence provide a useful comparison to hydrogen with systematic effects limited below the ppb level of precision. I think it would be helpful to include a projected precision in antihydrogen spectroscopic measurements based on anticipated antihydrogen production by the group. This would allow reader to gauge the value of the apparatus and what can be expected in the antimatter sector where this research will makes its first real contribution to physics though novel measurements.

This paper will be of interest to the large community of researchers that are following antihydrogen research at CERN although it does not hold significant value for the precision measurements community in physics as is. It makes well supported claims of the development and testing of the apparatus that are convincing to this reader.

The precision of the present measurements are limited by the statistical uncertainty in the measurement. The reader notes that the uncertainty in systematic effects is from the time standard and is included as a conservative limit in these effects. It would seem reasonable to reduce the statistical uncertainty to a similar level as large numbers of antihydrogen atoms will be much more difficult to achieve. Although this would be ideal, the tests of the apparatus confirm ppb measurements can be achieved with the apparatus. Again, the reader would like to see a projection of the required time to achieve a similar precision with expected antihydrogen production.

Although it seems obvious it is not clearly stated that the 1.6Hz shift (due to the 10MHz reference calibration) is corrected for or not to achieve the present agreement to past measurements. Under the assumption that the shift was corrected, no disagreement is observed with the past, more precise, measurements.

The claims are appropriately discussed in the context of antihydrogen research and precision tests of physics.

Reviewer #2 (Remarks to the Author):

The report "In beam measurement of the hydrogen hyperfine splitting - towards antihydrogen spectroscopy" by M. Diermaier et al. reports on a first demonstration of a high accuracy measurment of the hyperfine splitting in hydrogen using the ASACUSA antihydrogen apparatus. This is an important milestone in the direction of a high-presion meaurement of this quantity in antihydrogen.

The manuscript is well written and understandable for people that are not working in the field. Large sets of data have been collected to investigate systematic dependencies since the resonance line with its complicated structure has to be split to a level of 1E-5. The results are promising with respect to antihydrogen since the known splitting of normal hydrogen can be well reproduced within uncertainty. The uncertainty is three orders of magnitude larger than the best known value for the splitting from the MASER, but more than an order of magnitude more precise than the best Rabi-type in-beam measurements of this quantity. If a similarly accurate result for antihydrogen can be reached, this will represent a major improvement of CPT tests.

To this end, the manuscript fails to inform the reader how far the results achieved here can be transferred to antihydrogen. What are the expectations now for anti-hydrogen spectroscopy given the much lower production rates? According to the paper, the flow rate of hydrogen used in the experiment is huge (on the order of 1E17) while only a few 10-thousands of atoms will be available in the antimatter experiment. Can this all be compensated by the higher detection efficiency for antimatter? What is the expected linewidth for the two-dip structure when recorded with antihydrogen? Only if those points are at least briefly discussed, the reader can estimate how large a step towards antihydrogen spectroscopy this really is. This could be well inserted before the methods section since currently the main section ends somehow very abrupt with the error budget discussion.

Response to the Reviewers
reference number: NCOMMS-16-23561-T

First of all we wish to thank the Reviewers for their positive comments on our manuscript and the constructive criticism, which mainly concerns the lack of a discussion linking our results to antihydrogen hyperfine spectroscopy. Please find a point-by-point reply to the comments below, together with a description of the according changes.

> *The original reviews are indented and in italic fonts*

Our replies are interspersed.
Several comments by the two reviewers address the same issue. Instead of giving the same reply in different words, we copied such sections.

In the revised manuscript we have highlighted all changes:

- smaller changes highlighted in yellow
- complete new sections and figures enclosed in squares (instead of highlights for better readability)
- changes made in accordance with the *manuscript checklist* provided by the editor are highlighted in green

Reviewer #1 (Remarks to the Author):

> *I have been asked to review the article "In-beam measurements of the hydrogen hyperfine splitting – towards antihydrogen spectroscopy" for Nature Communications. Please find it here.*
> *The paper makes a number of well supported major claims about the research. A great deal of progress has been made to prepare for antihydrogen spectroscopy by the ASACUSA collaboration. As all experiments with antimatter atoms, it will be extremely challenging to achieve sufficiently high signal to noise ratios for precise spectroscopy due to the small number of atoms that can be produced, limited by antiproton availability. This warrants the preparation and testing of such apparatus with matter atoms wherever possible as described here. The apparatus has been developed over a long period and with a high level of care and planning to achieve its present state. The authors are applauded for this effort and congratulated on their progress in developing this novel apparatus for antihydrogen measurements.*
> *The work results in a comparison of spectroscopic measurements in hydrogen intervals at the ppb level with the much high precision (ppt and ppq level) achieved in apparatus not suited for antihydrogen spectroscopy and referenced within. The present results are in reasonable agreement and hence conclude that there are no systematic shifts evident at the ppb level. Although antihydrogen production will be with a very different technique and will result and many orders of magnitude*

reduced signal counts, these results will not be systematically shifted and hence provide a useful comparison to hydrogen with systematic effects limited below the ppb level of precision. I think it would be helpful to include a projected precision in antihydrogen spectroscopic measurements based on anticipated antihydrogen production by the group. This would allow reader to gauge the value of the apparatus and what can be expected in the antimatter sector where this research will makes its first real contribution to physics though novel measurements.

We added a Discussion section, where we start with a statement on the (first-stage) precision goal of ≤1 ppm for antihydrogen, as outlined in the ASACUSA proposal. Indeed it seemed necessary to clarify, that the ppb result achieved on hydrogen should not be interpreted as setting the goal for antihydrogen with the present apparatus and method to the same level.
In order to make a projection we use the hydrogen data to relate the precision of a resonance scan to experimental parameters (equation (5)). In a second step we use this relation together with assumptions - to the best of our knowledge - on the antihydrogen beam properties to estimate the required number of antihydrogen in order to reach the precision goal of ≤1 ppm (in this case the number of antihydrogen means the events registered at the annihilation detector = signal of Rabi spectroscopy measurement). We conclude that at least 8000 antihydrogen would be needed.
Technical details of the projection are treated in a new subsection of the Methods.

This paper will be of interest to the large community of researchers that are following antihydrogen research at CERN although it does not hold significant value for the precision measurements community in physics as is. It makes well supported claims of the development and testing of the apparatus that are convincing to this reader.
The precision of the present measurements are limited by the statistical uncertainty in the measurement. The reader notes that the uncertainty in systematic effects is from the time standard and is included as a conservative limit in these effects. It would seem reasonable to reduce the statistical uncertainty to a similar level as large numbers of antihydrogen atoms will be much more difficult to achieve.

It is true, that apart from the time standard, we could not find any hints for systematic effects. In fact, even the shift of the time standard cannot be concluded from the data but follows only from an independent calibration. At the achieved level of statistical uncertainty we can only put an upper bound for any other systematic effects. However, it is hard to imagine, that the same level of statistical uncertainty can be achieved with antihydrogen in the near future using the present apparatus and methods. Therefore, we don’t feel a strong need to reduce the statistical uncertainty of the hydrogen measurement with the present apparatus. A ppb or even ppt result on antihydrogen is more likely to be

achieved by more demanding methods. Their treatment we regard beyond the scope of this manuscript.

> *Although this would be ideal, the tests of the apparatus confirm ppb measurements can be achieved with the apparatus. Again, the reader would like to see a projection of the required time to achieve a similar precision with expected antihydrogen production.*

A statement on the time necessary to reach this goal will depend strongly on the progress within the ASACUSA collaboration. We therefore prefer to estimate the number of antihydrogen events required at the annihilation detector to reach ≤1 ppm precision instead of providing the required time. The characterization of the properties of the antihydrogen beam is an ongoing effort of utmost priority. As soon as reliable values on the beam properties are available the so far assumed properties can be replaced in the projection to yield a more reliable estimate.

> *Although it seems obvious it is not clearly stated that the 1.6 Hz shift (due to the 10 MHz reference calibration) is corrected for or not to achieve the present agreement to past measurements. Under the assumption that the shift was corrected, no disagreement is observed with the past, more precise, measurements.*

We agree, that this was not clearly stated. We now expand on this topic in the corresponding section to make clear, that we decided not to apply a correction for two reasons: a correction fully accounting for the shift found by the calibration would move the central value by 1.6 Hz, which is less than half of the statistical uncertainty and hence not significant. Moreover, the calibration was not done immediately after the measurement. Therefore it didn't seem evident, that the very same shift as found by the calibration was present during the measurement. As a consequence one would need to justify, which fraction of the shift should be applied. An assumption of linear evolution between a previous calibration would suggest <1 Hz shifts, which are statistically even less relevant. Linear interpolation would ignore down time of the Rb-clock as well as other effects, for instance by several transports. Consequently, we preferred to take the shift of 1.6 Hz as a systematic uncertainty. We note, that applying a shift would have resulted in better agreement of our result with the "maser-value".

> *The claims are appropriately discussed in the context of antihydrogen research and precision tests of physics.*

Reviewer #2 (Remarks to the Author):

> *The report "In beam measurement of the hydrogen hyperfine splitting - towards antihydrogen spectroscopy" by M. Diermaier et al. reports on a first demonstration of a high accuracy measurement of the hyperfine splitting in hydrogen using the ASACUSA antihydrogen apparatus. This is an important milestone in the direction of a high-precision measurement of this quantity in antihydrogen.*
> *The manuscript is well written and understandable for people that are not working in the field. Large sets of data have been collected to investigate systematic dependencies since the resonance line with its complicated structure has to be split to a level of 1E-5. The results are promising with respect to antihydrogen since the known splitting of normal hydrogen can be well reproduced within uncertainty. The uncertainty is three orders of magnitude larger than the best known value for the splitting from the MASER, but more than an order of magnitude more precise than the best Rabi-type in-beam measurements of this quantity. If a similarly accurate result for antihydrogen can be reached, this will represent a major improvement of CPT tests.*

From the reviewer's comment we felt the need to clarify, that the achieved ppb result on hydrogen should not be interpreted as setting the (first-stage) goal for antihydrogen with the present apparatus and method to the same level.
Therefore, we added a summary of our main conclusion at the end of the introduction (previously only the main result were summarized), where we mention the first-stage goal of the antihydrogen ground-state HFS experiment of ≤1 ppm, as outlined in the ASACUSA proposal. We also added our estimate on the number of antihydrogen atoms required for a 1 ppm measurement which amounts to 8000.
NB: the addition of a summary on the conclusions at the end of the introductions is also motivated by the *manuscript checklist.*

> *To this end, the manuscript fails to inform the reader how far the results achieved here can be transferred to antihydrogen. What are the expectations now for anti-hydrogen spectroscopy given the much lower production rates?*

We added a Discussion section, where we start with a statement on the precision goal of ≤1 ppm for antihydrogen. In order to make a projection we use the hydrogen data to relate the precision of a resonance scan to experimental parameters (equation (5)). In a second step we use this relation together with assumptions - to the best of our knowledge - on the antihydrogen beam properties to estimate the required number of antihydrogen in order to reach the (first-stage) precision goal of ≤1 ppm (in this case the number of antihydrogen means the events registered at the annihilation detector = signal of Rabi spectroscopy measurement). As mentioned before, we conclude that at least 8000 antihydrogen would be needed.

Technical details of the projection are treated in a new subsection of the Methods.

According to the paper, the flow rate of hydrogen used in the experiment is huge (on the order of 1E17) while only a few 10-thousands of atoms will be available in the antimatter experiment. Can this all be compensated by the higher detection efficiency for antimatter? What is the expected linewidth for the two-dip structure when recorded with antihydrogen?

The flow rate stated in the manuscript is calculated from the regulated H_2-flow into the dissociation plasma tube. Only a fraction of the generated H atoms arrives at the detector due to the differential pumping used. Combined with the detection efficiency of the Q-mass spectrometer (~10E-8) the rate of detected hydrogen atoms goes down to the mentioned level of tens of kHz (also shown in figure 3 or listed in table "Parameters of the data sets").
We have modified the last paragraph of the subsection "Experimental setup" slightly in order to clarify this point.

The line width is inversely proportional to the interaction time (i.e. for a given cavity length, proportional to the velocity). The characteristic velocities of the envisaged 50 K antihydrogen beam are in the region of the observed hydrogen velocities. Hence, similar line widths are expected.
For antihydrogen, a detection efficiency close to unity and a better signal-to-background ratio will partly compensate for the much smaller count rate. As now discussed a precision on the antihydrogen HFS, worse by ~3 orders of magnitude compared to the present hydrogen HFS value should be achievable with around 8000 detected antihydrogen events.

Only if those points are at least briefly discussed, the reader can estimate how large a step towards antihydrogen spectroscopy this really is. This could be well inserted before the methods section since currently the main section ends somehow very abrupt with the error budget discussion.

The discussion of the precision goal for antihydrogen and a projection have been added at the suggested section of the manuscript.

REVIEWERS' COMMENTS:

Reviewer #1 (Remarks to the Author):

The authors addressed my minor concerns and I am satisfied that this paper, in this updated form, should be published

Reviewer #2 (Remarks to the Author):

All issues addressed in the previous referee reports have been addressed in-depth. It is now much clearer what accuracy is envisaged in the antihydrogen spectroscopy. I agree that it is preferred to estimate the number of Hbar events that have to be detected instead of the time required to get to the targeted precision.
